# Health-related quality of life of subjects with Barrett's esophagus in a Chinese population

**DOI:** 10.1371/journal.pone.0190201

**Published:** 2017-12-21

**Authors:** Shou-Wu Lee, Han-Chung Lien, Chi-Sen Chang, Chung-Wang Ko, Chun-Fang Tung, Hong-Zen Yeh

**Affiliations:** 1 Division of Gastroenterology, Department of Internal Medicine, Taichung Veterans General Hospital, Taichung, Taiwan; 2 Department of Internal Medicine, Chung Shan Medical University, Taichung, Taiwan; 3 Department of Internal Medicine, National Yang-Ming University, Taipei, Taiwan; University Hospital Llandough, UNITED KINGDOM

## Abstract

**Aim:**

The aim of this study was to investigate health-related quality of life (HRQoL) of a Chinese population with Barrett's esophagus (BE).

**Methods:**

Data from subjects with BE from a single hospital were prospectively collected from October 2012 to December 2014. The exclusion criteria included total esophagectomy, severe cardiopulmonary deficiency, malignancy, or other unsuitable conditions for scope. All the enrolled cases were asked to complete the Reflux Disease Questionnaire (RDQ), the short form-12, (SF-12), and the Hospital Anxiety and Depression Scale (HADS).

**Results:**

In total, 139 subjects were enrolled, and the mean age of the cases was 61.85 years old. Most subjects had short-segment BE (SSBE) (92.8%) and non-dysplastic BE tissue (94.2%). The mean physical and mental composite scores, PCS and MCS, of SF-12 were 44.14 and 45.53, respectively. The SF-12 scores in BE individuals were similar in men and women, elderly and non-elderly, LSBE and SSBE, coexisting EE and no-EE, and dysplastic and non-dysplastic. The appearance of reflux symptoms tended to decrease SF-12 scores in affected individuals, especially heartburn. The rates of anxiety and depression accounted for 25.2% and 17.3% of these cases, respectively.

**Conclusion:**

Our study found HRQoL in BE patients was strongly associated with presentation of reflux symptoms.

## Background

Barrett's esophagus (BE) is defined as the appearance of intestinal metaplasia (IM) of the esophageal squamous epithelium, and is considered to be a complication of gastroesophageal reflux disease (GERD) [1]. Symptoms of GERD, such as heartburn or acid regurgitation, have been associated with an increased risk of BE [2,3]. According to a previous study, individuals with BE reported worse health-related quality of life (HRQoL) compared with that of the general population [4]. Additionally, BE patients might have a higher possibility of anxiety or depression compared with the general population [5]. Because the increased prevalence rate of GERD in the East countries, BE might become a important disease in Asian population in the nearly future. However, there are limited data on the quality of life in Chinese BE individuals. The aim of this study was to investigate HRQoL in a Chinese population with BE.

## Methods

This study was approved by Institutional Review Board of the Taichung Veterans General Hospital (No. CF14040). Data from subjects with BE who visited the Medical Screening Center at Taichung Veterans General Hospital were prospectively collected from October 2012 to December 2014. The general data of enrolled patients, including age, gender, body mass index (BMI), and waist circumference were recorded. All patients underwent an open-access transoral upper gastrointestinal (UGI) endoscopy, which was performed using a high-resolution endoscope with white light and narrow band imaging (NBI), and four-quadrant tissue biopsies were obtained as per AGA recommendations [6]. BE was diagnosed by the typical IM pattern, and all biopsies were obtained above of esophagogastric junction (EGJ). The endoscopic findings, including erosive esophagitis (EE), short segment BE (SSBE, extending <3cm into the esophagus) or long segment BE (LSBE, extending ≧3cm into the esophagus), and pathologic dysplastic appearance of BE tissue, were collected. The exclusion criteria included total esophagectomy, severe cardiopulmonary deficiency, malignancy, other unsuitable conditions for UGI endoscopy, or segments of metaplastic columnar epithelium <1 cm, which classified as “specialized IM of the EGJ”.

All the enrolled cases were asked to complete questionnaires to evaluate reflux symptoms (Reflux Disease Questionnaire, RDQ), generic HRQoL (short form-12, SF-12, Chinese version), and status of anxiety or depression (Hospital Anxiety and Depression Scale, HADS). RDQ is a 12-item self-administered questionnaire designed to assess the frequency and severity of heartburn, regurgitation, and dyspeptic complaints [7]. The severity of heartburn or acid regurgitation of each individual was classified as nil (average scores 0), mild (average scores 1–2), or severe (average scores 3–4). The SF-12 is a multipurpose short-form survey with 12 questionnaires, all selected from the SF-36 health survey, comprising two scales that provide information on the patient’s physical and mental functioning (physical health composite scale scores, PCS; mental health composite scale scores, MCS) [8]. The Chinese Version was obtained by translation, validation, and normalization [9]. HADS is a self-assessment scale which has been shown to be a reliable instrument for detecting states of depression and anxiety in the setting of a hospital medical outpatient clinic [10]. Scores of <7 are normal, whereas scores of 8 to 10 and >10 represent possible and definite presentation of anxiety or depression, respectively.

Data are expressed as standard deviation of the mean for each of the measured parameters. Endoscopic patterns, pathologic findings of BE tissue, and presentation of reflux symptoms of each stratified group are expressed as a percentage of the total patient number. Statistical comparisons were made using independent t test to analyze SF12 scores in the subgroups. A p-value below 0.05 was considered statistically significant.

## Results

A total of 139 subjects were enrolled in our study. The baseline characteristics as well as endoscopic and pathologic presentations of BE are displayed in Table 1. The mean age of these cases was 61.85 years old, and the average BMI and waist circumference were 24.18 kg/m2 and 86.56 cm, respectively. There were 96 men (69.1%) and 43 women (30.9%) in our study. The endoscopic and pathologic patterns of BE showed most of our cases were SSBE (92.8%), without coexisting EE (68.8%), and had non-dysplastic BE tissue (94.2%).

**Table 1:** The baseline characteristics of enrolled individuals with Barrett's esophagus.

		Overall cases (N = 139)		
		M ± SD	N	%
Age (years)		61.85 ± 15.49		
BMI (kg/m2)		24.36 ± 3.55		
Waist circumferences (cm)		88.20 ± 8.71		
Gender	Male		96	(69.1%)
	Female		43	(30.9%)
BE length	LSBE		10	(7.2%)
	SSBE		129	(92.8%)
ECJ	EE		42	(30.2%)
	No-EE		97	(69.8%)
BE pathology	Dysplastic		8	(5.8%)
	Non-dysplastic		131	(94.2%)

Abbreviations: BE, Barrett’s esophagus; BMI, body mass index; ECJ, esophagocardiac junction; EE, erosive esophagitis; LSBE, long-segment Barrett’s esophagus; M, mean; N, numbers; SD, standard derivation; SSBE, short-segment Barrett’s esophagus

The results of the questionnaires are shown in Table 2. In the RDQ, among patients who reported heartburn, symptoms were nil, mild, and severe in 75.5%, 18.7%, and 5.8%, respectively. For the acid regurgitation dimension of the RDQ, the proportions of nil, mild, and severe symptoms were 48.2%, 37.2%, and 14.4%, respectively. The mean PCS and MCS of the SF-12 were 44.14 and 45.53, respectively. The evaluation of mental health by HADS showed that the rate of possible or definite anxiety and depression among these cases accounted for 25.2% and 17.3% of the patients, respectively.

**Table 2:** The results of RDQ and HADS questionnaires.

	Overall cases (N = 139)		
	M ± SD	N	%
RDQ			
nil HB		105	(75.5%)
mild HB		26	(18.7%)
severe HB		8	(5.8%)
nil AR		67	(48.2%)
mild AR		52	(37.4%)
severe AR		20	(14.4%)
SF-12			
PCS	44.14 ± 9.42		
MCS	45.53 ± 10.74		
HADS			
Anxiety		35	(25.2%)
Depression		24	(17.3%)

Abbreviations: AR, acid regurgitation; HADS, Hospital anxiety and Depression scales; HB, heartburn; MCS, mental health composite scale scores; M, mean; N, numbers; PCS, physical health composite scale scores; RDQ, Reflux Disease Questionnaire; SD, standard derivation; SF-12, short form-12.

As shown in Table 3, the SF-12 scores in the subgroups all showed non-significant differences, as follows: men (PCS 44.24, MCS 46.13) vs. women (PCS 43.89, MCS 44.19), elderly (≧65 years-old, PCS 43.58, MCS 44.90) vs. non-elderly (<65 years-old, PCS 44.52, MCS 45.98), LSBE (PCS 44.32, MCS 41.10) vs. SSBE (PCS 44.12, MCS 45.88), dysplastic (PCS 46.77, MCS 43.45) vs. non-dysplastic (PCS 43.98, MCS 45.66), coexisting EE (PCS 42.22, MCS 43.75) vs. non-EE (PCS 44.87, MCS 46.31).

**Table 3:** The scores of SF-12 questionnaire in each subgroup.

		N	PCS	*P-value*	MCS	*P-value*
			M ± SD		M ± SD	
Gender	Male	96	44.24 ± 9.52	0.840	46.13 ± 11.06	0.326
	Female	43	43.89 ± 9.29		44.19 ± 9.97	
Age	Elderly^#^	57	43.58 ± 9.22	0.563	44.90 ± 10.91	0.562
	Non-elderly^#^	82	44.52 ± 9.59		45.98 ± 10.66	
BE length	LSBE	10	44.32 ± 10.99	0.956	41.10 ± 12.28	0.176
	SSBE	129	44.12 ± 9.33		45.88 ± 10.58	
BE pathology	Dysplastic	8	46.77 ± 6.54	0.286	43.45 ± 12.23	0.632
	Non-dysplastic	131	43.98 ± 9.56		45.66 ± 10.68	
ECJ	EE	42	42.44 ± 10.08	0.162	43.75 ± 11.49	0.198
	Non-EE	97	44.87 ± 9.07		46.31 ± 10.38	

Abbreviations: BE, Barrett’s esophagus; BMI, body mass index; ECJ, esophagocardiac junction; EE, erosive esophagitis; LSBE, long-segment Barrett’s esophagus; M, mean; MCS, mental health composite scale scores; N, numbers; PCS, physical health composite scale scores; SD, standard derivation; SSBE, short-segment Barrett’s esophagus. P-values were all analyzed with independent t test

^#^Definition of elderly: age≧65 years-old; non-elderly: age<65 years-old.

**Fig 1:**
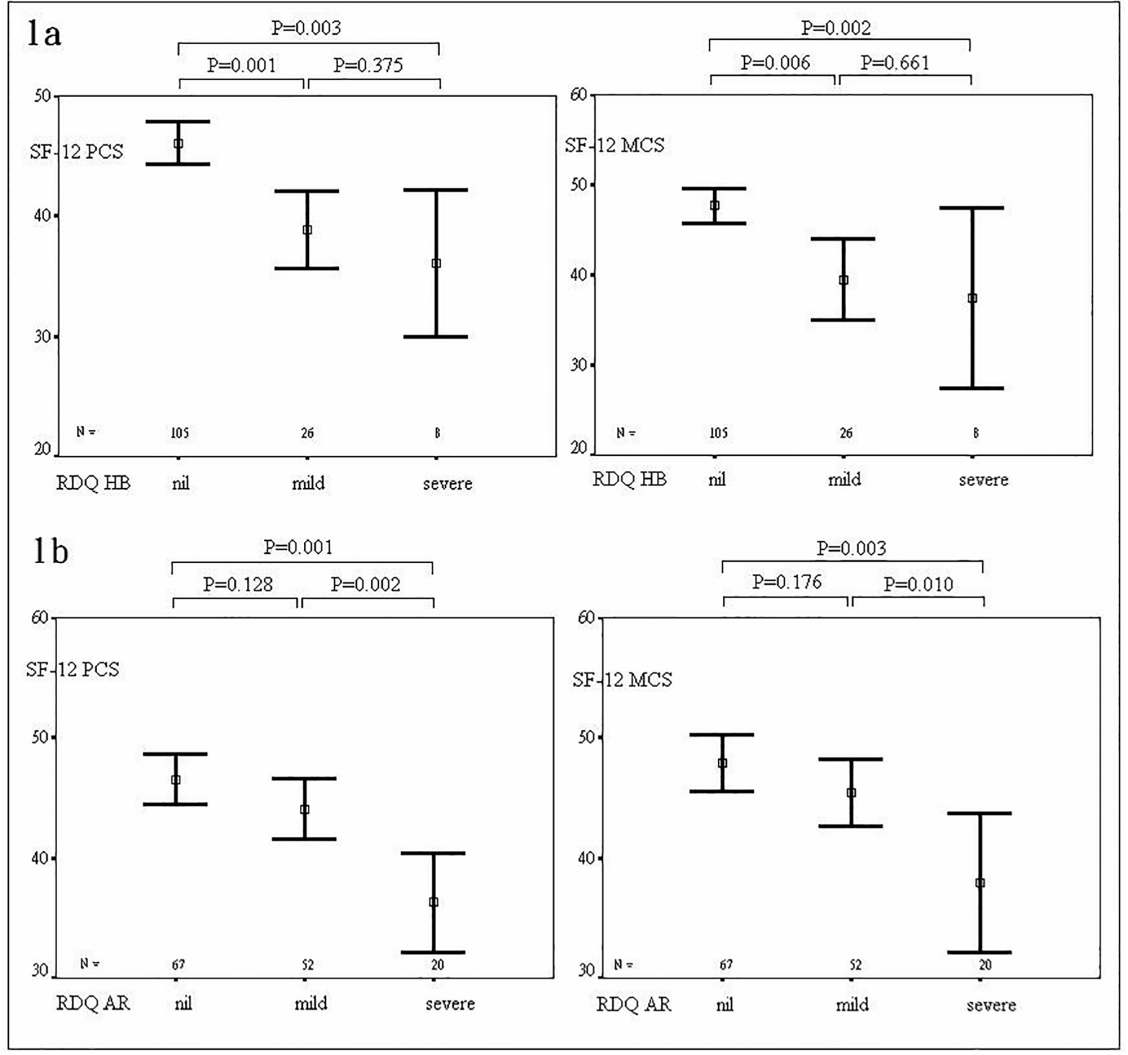
SF12 scores of the subgroups of heartburn (1a) and acid regurgitation (1b) symptom severity.

The SF-12 scores of the individuals with different GERD symptom severity are displayed in Fig 1. In the nil, mild, and severe heartburn symptom subgroups, the PCS were 46.06±9.11, 38.88±7.98, 36.02±7.30, respectively, and for MCS they were 47.65±9.71, 39.47±11.19,37.43±12.01, respectively. The differences between the nil and mild symptom, or nil and severe symptom, were significant. In the nil, mild, and severe acid regurgitation symptom subgroups, PCS and MCS were 46.55±8.68, 44.04±9.05, 36.28±8.79, and 47.90±9.70, 45.41±10.12, 37.93±12.44, respectively. There were significant differences between nil and severe symptom, or mild and severe symptom.

**Fig 2:**
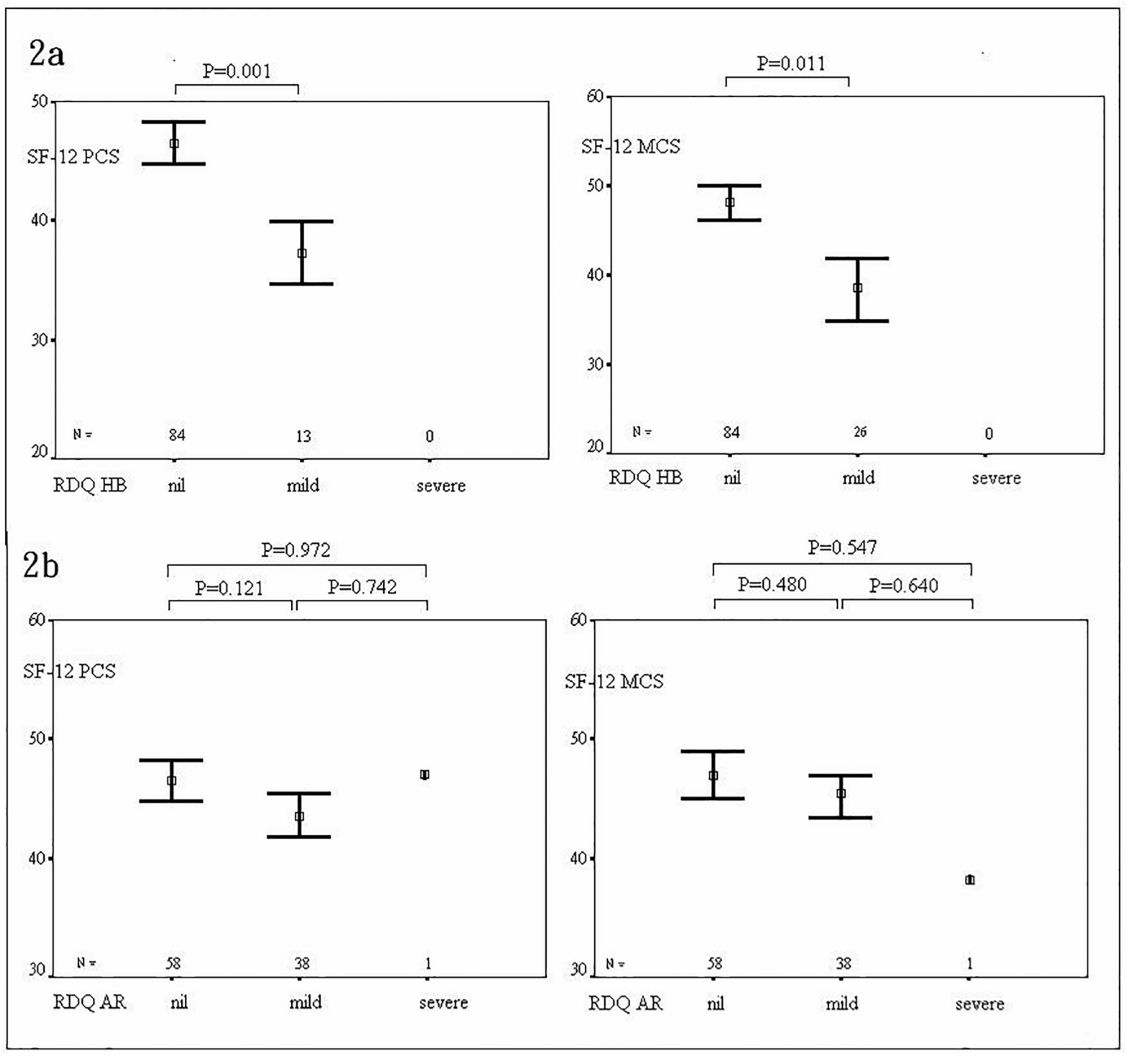
SF12 scores of the subgroups of heartburn (2a) and acid regurgitation (2b) symptom severity after individuals with anxiety or depression were excluded.

The association between HRQoL and the HADS results are shown in Table 4. The BE patients with anxiety (mean PCS 38.42 vs. 46.06, mean MCS 38.89 vs. 47.77) and depression (mean PCS 39.38 vs. 45.13, mean MCS 40.51 vs. 46.58) had lower SF-12 scores, both in physical and mental functioning. As shown in Fig 2, the association of SF-12 scores and GERD symptom severity was rechecked after excluding the cases with anxiety or depression. In the nil and mild heartburn symptom subgroups, PCS were 47.71±8.44 and 38.46±7.40, respectively, and for MCS, they were 49.52±8.33 and 39.17±12.27, respectively. The differences were significant. In the nil, mild, and severe acid regurgitation symptom subgroups PCS and MCS were 47.65±8.15, 44.64±9.78, 47.94, and 48.65±9.57, 47.22±9.70, 38.64, respectively. The differences between these subgroups were non-significant.

**Table 4:** The association of quality of life and HADS questionnaire results.

		N	PCS	*P-value*	MCS	*P-value*
			M ± SD		M ± SD	
HADS	Anxiety	35	38.42 ± 8.60	0.001	38.89 ± 10.59	0.001
	Non-anxiety	104	46.06 ± 8.92		47.77 ± 9.86	
	Depression	24	39.38 ± 7.61	0.006	40.51 ± 10.33	0.011
	Non-depression	115	45.13 ± 9.48		46.58 ± 10.56	

Abbreviations: HADS, Hospital anxiety and Depression scales; MCS, mental health composite scale scores; M, mean; N, numbers; PCS, physical health composite scale scores; SD, standard derivation. P-values were all analyzed with independent t test

## Discussion

Barrett's esophagus (BE) is defined as a metaplastic change from squamous epithelium to columnar epithelium in the distal esophagus, and is considered to be a pre-malignant disease [1]. According to previous studies, BE showed a male predominance [11], and most BE in Asian countries was reported to be short segment BE (SSBE) [12]. Our enrolled patients had a similar distribution.

Chronic presentation of GERD is considered a risk factor of BE, and BE presents in a higher percentage in patients with reflux symptoms than in those without reflux [1,6]. According to previous reports, the prevalence of BE was between 1% and 2% in population-based studies, but in patients with GERD symptoms, the prevalence of BE ranged from 10% to 18% [12,13]. However, approximately 40% of BE cases were without any reflux symptoms [12,14]. Among our cases, the prevalence rates of enrolled patients without heartburn and acid regurgitation symptoms were, 75.5% and 48.2%, respectively. This result implies GERD symptoms might play a minor role in the early detection of BE in a Chinese population.

In two previous studies involving the evaluation of HRQoL in Germany and the United States, PCS scores were 41.8 to 42.6, and MCS scores were 46.2 to 51.7%, respectively [4,15], which was similar to the results of our study. Moreover, the US study revealed that BE patients had better generic and disease-specific quality of life than that of GERD patients [4]. However, another study indicated no significant difference between BE and GERD cases [16].

A recent study enrolling 84 BE cases in Taiwan found BE had significantly poor HRQoL, based on WHOQOL-BREF scores, compared with healthy controls. The difference existed in the physical domain, but not in the social or psychological domains [17]. However, the HROoL of each subgroup of BE patients, or the severity of coexisting GERD symptoms, were not evaluated in the above studies.

In our cases, there were similar presentations of HRQoL in BE individuals based in gender (men vs. women), age (elderly vs. non-elderly), BE length (LSBE vs. SSBE), coexisting EE (EE vs. no-EE), and pathologic patterns (dysplastic vs. non-dysplastic). A significant positive association between GERD symptom severity and HRQoL was noted in our study. The SF-12 scores started to drop once the BE patients developed the symptom of heartburn, even when the symptom severity was mild. In contrast, the negative impact of acid regurgitation on the SF-12 scores only occurred in BE patients with severe acid regurgitation.

BE patients were considered to have a high risk of anxiety compared with the general population [5], although another study reported only minimal depression and anxiety in BE cases [18]. Our results demonstrated a high rate of anxiety and depression in BE individuals. Due to the negative impact of anxiety or depression on HRQoL, we excluded these cases and reassessed the data. The influence of heartburn on quality of life in BE cases remained.

There were several limitations in our study. First, this study was a hospital-based investigation in a single tertiary care center. Selection bias might have existed and thus the patient population may not have been representative of the general population. Second, use of anti-secretory agents for reflux symptoms was not determined. The rate of GERD may therefore have been underestimated. Thirdly, there is no control group in our study, which making the baseline ratio of anxiety/depression or HRQoL hard to determine. Finally, the questionnaires of reflux, HRQoL, anxiety or depression symptoms that were completed by the enrolled individuals were self-reported, and therefore some errors might have occurred. Further community-based research with more variables is needed.

In conclusion, our study found HRQoL in BE patients was strongly associated with presentation of GERD symptoms, especially heartburn. Coexisting anxiety and depression was also highly prevalent in BE individuals.
